# ‘South-Working’: Return mobilities and remote work during COVID-19

**DOI:** 10.1177/0308275X241299327

**Published:** 2024-12-09

**Authors:** Flavia Cangià

**Affiliations:** Université de Fribourg, Switzerland

**Keywords:** career, digital remote work, migration, pandemic, return mobilities

## Abstract

This article explores the experiences of professionals who, prompted by the pandemic, returned to their hometowns in Italy while continuing to work remotely, a trend known as ‘South-Working’. I explore how the pandemic changed these individuals’ usual mobility routines and led them to return home and reconsider life priorities, all while leveraging digital remote work and new mobility strategies. I draw upon ethnographic fieldwork, including online video interviews, observation in social media platforms and visits to co-working spaces across the northern part of Sicily. This article contributes to the anthropology of (im)mobility by exploring the transformative impact of the pandemic and the digital on mobility regimes and the boundaries associated with movement, work and life. It challenges established categories and normative temporalities of (im)mobilities, offering a new perspective on the evolving dynamics of work and mobility in the digital era, and on the transformation of the meaning of ‘essential’ and ‘non-essential’ mobilities.

In the wake of Covid-19, the transition to digital work rendered certain work-related mobilities, such as business travel and commuting, non-essential (Cook, 2020). It also encouraged alternative mobile practices that held significance at more personal level (Salazar, 2021a). Prompted by the new access to and acceptance of remote work, some people considered travelling to – and working remotely from – distant locations (Hermann and Paris, 2020). For others, return, whether permanent or temporary, appeared possible. The opportunities of remote work during the pandemic led to a unique occurrence – people returning to Italy or to their hometowns from other Italian regions, and choosing to work far from their usual workplace during the critical phase of the pandemic, and some even after that. This article aims to explore the experiences of some of these returnees. This phenomenon, referred to as ‘South-Working’, held special significance for me, an Italian-origin migrant living in Switzerland, as my extended family was in Italy throughout the crisis. Contemplating the possibility of making a similar choice to return, even in an imaginative sense, inspired my curiosity: this ‘imaginative return’, where I mentally explored the idea of returning to my hometown, prompted my interest in exploring the implications inherent in these return mobilities. A focus on these mobilities contributes to understanding the broader implications of mobility and digitalism^1^ in the post-pandemic era.

This article contributes to theoretical debates in anthropology on (im)mobility (Glick Schiller and Salazar, 2013), with a special focus on the transformative impact of the pandemic and the digital on mobility regimes and the boundaries associated with movement, work and life. It challenges established categories and normative temporalities of (im)mobilities, offering a new perspective on the evolving dynamics of work and mobility in the digital era, and on the transformation of the meaning of ‘essential’ and ‘existential’ mobilities (Salazar, 2021a). I look at how the digital re-shapes the traditional boundaries of movement, work and life, challenging established anthropological categories and the normative temporalities of (im)mobilities (Amit and Salazar, 2020), especially in an era where remote work has become socially more accepted (Cook, 2020). I explore the interplay of return and remote work through the lens of ‘existential mobility’(Hage, 2005). This approach offers a richer understanding of how people negotiate mobilities through the digital, and the profound existential shifts that these experiences can trigger. It expands the anthropological theoretical knowledge of ‘existential mobility’, shedding light on how these mobile professionals reconsider normative understandings of when and why mobility is essential or non-essential. In the next section, I highlight the contributions of my research and I delve into the concepts of return and existential mobilities.

## Return, remote work and existential mobility

Understanding how the pandemic has influenced decisions of return, especially for those people with a newfound ability to work remotely, remains somewhat limited across the social sciences (Mencutek, 2022). Yet the potential transformations brought about by the rise of digitalization in the post-pandemic era, coupled with physical mobilities, career trajectories, and work–life balance, are subjects of increasing significance in envisioning the future of work and mobility (Adey et al., 2021). My research aims to fill this gap and to contribute to anthropological theory on mobility by understanding how digitalism, characterized by the widespread integration of digital technologies into various aspects of life, challenges the separation of categories and dimensions of mobility^2^ (Glick Schiller and Salazar, 2013; Bloch, 2020; Salazar, 2021a).

I critically reflect upon the unidirectional sense associated with return (King and Christou, 2011). I draw upon a broad definition of return mobilities (Long and Oxfeld, 2004). This definition includes return migration, as ‘the physical relocation of the migrant with the intention of staying for some time, maybe permanently, in the place of origin’ (King and Christou, 2011: 452) or a ‘reconstructed homeland (a particular site in the home country where one has never actually lived)’ (Long and Oxfeld, 2004: 4). It also includes return more broadly, referring to ‘forced’ repatriation, imagined or provisional return, such as various short-term visits, habitual, circular, or periodic movements (Long and Oxfeld, 2004). Return is not a one-way journey, but a cyclical process involving multiple forms of im/mobilities between various places. It stretches ‘its meaning across time, space and generations, and … the “place” of return and the type of movement can have various expressions – real, virtual, imagined, desired, forced or denied’ (King and Christou, 2011: 453). The phenomenon known as South-Working illuminates the contradictions of return to and from once-familiar environments that may then be permeated with elements of unfamiliarity and remoteness (Schuetz, 1945). It highlights a sense of potential for transformation, intertwined with movement, relationships, places, as well as the interplay between *roots* and *routes* (Clifford, 1997), between staying, belonging and moving. This article invites theoretical reflection on the categorization of mobile work and leisure practices as separate (Cook, 2022), and on the existential dynamics of mobilities and work in the digital age. I reflect on our understanding of place and its interplay with mobility and immobility. Place-making, in these return experiences, comes to include the displacement of the workplace to online spaces, on the one hand and, on the other, physical immobilization in a place of residence, in strong connection to the local community, family and a sense of belonging (Salazar, 2023).

I explore the interplay of return and remote work through the lens of ‘existential mobility’, with a focus on work. The concept of ‘existential mobility’, defined as the feeling that life moves on (Hage, 2005), delves into the deeper, existential implications of mobility (Salazar, 2021a), emphasizing how mobility is not just a physical act but also a fundamental component of how people construct and make sense of their lives (Cangià, 2023a; Salazar, 2018). This existential dimension emphasizes the transformative imaginary associated with mobility and its intersections with individuals’ subjectivities. Here, the concept of existential mobility sheds light on how return journeys, coupled with remote work, can be viewed in a perspective that goes beyond the short-term practical implications of the pandemic (family care, health and safety, financial considerations). It emphasizes the long-term transformations in individuals’ sense of belonging, their connection to place, and the re-evaluation of their life paths. Examining return from the perspective of existential mobility, in other words, elucidates the complex shift in the perception of mobility forms classified as ‘essential’ versus those seen as ‘existential’: ‘a situation such as the one surrounding the COVID-19 pandemic … shows that a demanding situation can lead to reflections on and changed understandings of what is commonly accepted as essential and existential mobility’ (Salazar, 2021a: 4). ‘Essential mobilities’ can be understood as those movements necessary for basic needs and survival, often linked to economic or career-oriented goals. Salazar rightly acknowledges that ‘individual understandings of which mobilities are essential can be quite different from the officially sanctioned categories’ (Salazar, 2021a: 12). The synergy between return mobilities and existential mobility can be observed through the experiences illustrated in this study: in the aftermath of the pandemic, individuals may reflect on the significance of the career-oriented mobilities that motivated their relocation in the first place; they may consider alternative forms of mobility, opting for a return to more life-oriented choices, to distant and at times remote places. The remoteness of certain places from the office and yet the proximity of these very places to other aspects of life are valued by participants as contributing to a ‘better life’. The remoteness and proximity of places at the spatial, relational, and temporal levels represent two important parameters for (re)imagining the course of life and mobilities are paced accordingly (Cangià, 2023b). The emotional attachment to the ‘South’ emerged as a result of experiencing the pandemic as a significant event that shook the ‘normal’ course and rhythm of life (Thomson et al., 2002). Respondents found solace in their homeplace, a town, a neighbourhood or, broadly, Italy, considering it ‘the best place’ to be during the critical moments of the pandemic.

I now present the South-Working project and my fieldwork.

## Return to the ‘South’ and remote work

The phenomenon of return to Italy certainly existed long before the pandemic, but it has intensified following new remote-working policies in some professional domains. During the pandemic, an increasing number of people engaged in remote work and returned, with the idea of reconnecting with spheres of life alternative to work (Celata, 2022). I like to keep the term proposed by the non-profit association called South-Working to describe this phenomenon: ‘South-Working’ – in Italian, *Lavorare dal Sud*. The association was formed in March 2020 by a group of young professionals and students united by their common condition of living far away from their hometowns in Italy during the pandemic and wanting to return. The term ‘South-Working’ in English plays with the idea of ‘smart-working’, which in the Italian context often refers to the flexibility of digital remote work. By replacing ‘smart’ with ‘South’, the term suggests that agile and remote work can be carried out effectively from a place that is considered ‘home’, rather than being from multiple (unknown) destinations. The flexibility inherent in ‘smart-working’ seamlessly aligns with various forms of mobility, including tourism, digital nomadism and hypermobile travels, leading to alternative destinations beyond traditional work boundaries (Green, 2020). ‘South’, as originally intended by the association, refers primarily to the southern part of Italy, but came to include other regions of Italy for those who returned from abroad. During their interview, one of the association’s founders articulated the notion of ‘South’ as a ‘relative’ concept, emphasizing that ‘we are always South to some other place’. Using the term ‘South’ here helps to clarify anthropological categories. On the one hand, it captures the complexity of return to southern Italy, acknowledging the socio-economic challenges in specific regions; at the same time, it also recognizes the broader, more abstract, idea of ‘Italy’ as ‘South’ in relation to some other places, as portrayed by some respondents, who highlighted the precarious and socio-economically challenging work conditions in Italy, compared to other countries. On the other hand, the term ‘South’ also signifies the potential within familiar settings, transforming them into destinations where work and leisure can be combined, while remaining open to the possibilities of individual or community change. It underscores the importance of digitalism and return in reshaping the boundaries between work and life: remote working is not ‘smart’ because it can be done anywhere, but it is ‘agile’ because it can integrate and people’s personal life and career, and make it easier to move between them.

The idea of South-Working may indicate inequalities in people’s capacity to return and work remotely, and in mobility and other infrastructural conditions between regions (Mirabile and Militello, 2022). Some are more conducive to remote work due to better internet connectivity, and the availability of social and economic resources, and overall infrastructure. In contrast, other regions face challenges in providing these essential elements for effective remote work. In this regard, the association South-Working launched a project across the whole country, to promote remote-working practices and advocate for the development of mobility infrastructures and internet connectivity in areas where these are still limited. The so-called *presidi di comunità* (community hubs) were promoted by the association, especially in small villages of southern Italy, but also in other regions, to attract remote workers and stimulate encounters with local communities. These are working spaces that offer a space to work and aim to foster the development of communities in terms of human capital with the return of skilled migrants (Mariotti et al., 2023).

The meaning of South-Working transcends mere geographical coordinates, representing an existential return to one’s *roots*, as well as an active participation in family life, or even in the rebuilding of one’s place of origin at a multifaceted level – be it in terms of social connections, cultural heritage, or economic vitality. The reciprocal relationship between personal and community transformation, facilitated by the return, sheds light on the shifting significance of mobilities that were once deemed non-essential. These mobilities now become essential for both individuals and local communities. The transformative potential for these people is connected to the prospect of working in improved life conditions, to a deep sense of being in motion on various levels – existential and social – as well as to the idea of contributing to the social and economic development of a place considered as home.

I conducted ethnographic fieldwork between October 2021 and August 2022, including online video interviews, observation and engagement in online social media platforms, and visits to some co-working spaces across the northern part of Sicily. In May 2021, I contacted the non-profit association South-Working, which introduced me to the case of Castelbuono as one of the earliest examples of a community hub. Castelbuono is located in the Madonie Mountains, one hour’s drive from Palermo. It is the first location where the South-Working project was implemented, with the construction of co-working spaces and other activities to facilitate the mobility and connectivity of workers. I visited the locales of the two co-working spaces in Castelbuono, respectively in the old castle and in the cloister of San Francesco, and conversed with some workers and one of the founders of the local project.

The South-Working association gave me access to its Facebook community. I also contacted other Facebook groups, such as *Torno in Italia* and *Bentornati al Sud*. I explored these groups and followed online conversations among members concerning their feelings and opinions regarding return and remote work. I introduced myself and the goals of my research on these online groups, and launched a call for participants, for those who recently returned during the Covid-19 pandemic and who work remotely. Some participants were recruited through these Facebook pages. Others through snowballing and personal contacts. The narrations of people active on these social media platforms are ethnographically relevant for they provide in-depth insights into the experiences of individuals engaged in digitalism in various forms.

Online video interviews were conducted with a total of 15 individuals (six men and nine women) with ages ranging from 22 to 45. Participants were employed in various fields, including IT, communication, academia, translation, education, digital marketing, and consumer services. Only two have children. Ten were in a relationship or married. I asked them about their experiences of the lockdown and the return. We talked about the challenges and benefits of returning and working remotely; the arrangements relating to career and life resulting from the remote work; their migration trajectories (reasons for returning, relocation process, previous experiences of migration, previous life before return, present situation, and future plans), everyday work practices, feelings about their distance from the workplace, workspace arrangements (e.g. home, office, co-working space) and work-related mobilities.

Respondents had returned from abroad or to their hometowns from other regions in Italy, from March 2020 onwards, and were working remotely. Nine people had returned to Italy from a foreign country. Five had moved from one Italian region to another. One respondent moved back to Italy but to a place other than her birth-town, and another did the same after staying for a few months in her native town. Some returned during the initial lockdown phase and continued without long breaks throughout 2020, and sometimes even beyond. Following the strictest lockdown measures, some alternated between periods of remote work from their hometowns, particularly during holidays, and other periods spent at the office. Others came back only during the lockdown and went back to their regular workplace at the end of it. However, they still had the option to work remotely occasionally, leading to increased travel to their hometowns over weeks and days. Others had shorter periods of both office work and remote work. One respondent, at the time of their interview, was about to leave again after one year in Italy, to go back to the previous migration destination.

All worked remotely during the time of the crisis and mostly after that, until the time of the interview. The majority continued working for the same employer. Two of them lost their jobs briefly after returning but continued working remotely for a few months. One respondent, after an initial phase of return right after the lockdown period, returned to the office, but could negotiate periods of remote work with the employer and would travel to the workplace more often whenever necessary.

For some, remote work represented a new experience during the lockdown. Except for one respondent, they all enjoy being able to work this way and wanted to continue doing so. Distance from their place of work and co-workers contributed to maintaining concentration and using breaks to focus on personal interests or family issues. Some proactively engaged in discussions with their employers to explore the option of continuing remote work even beyond the pandemic. A few people simply continued following employers’ instructions and working from home. Some managed to negotiate a new professional profile in the company, or a different form of contract with their employer.

I shared my own experience as a migrant in Switzerland. My experiential and emotional affinities with these people’s stories, as well as my personal feelings regarding being distant from part of my family when the pandemic hit, facilitated exchanges on these topics (Forsey, 2010). I came across the South-Working association while reading the newspaper shortly after the lockdown started, and I was deeply moved by the choice of these individuals. Their choice resonated with the feelings I experienced at the beginning of the pandemic, as an Italian-origin migrant who moved to Switzerland for work years ago, while my extended family remained in Italy. Before the pandemic, family visits represented a habitual part of my life. The realization that some of my ‘essential’ mobilities had come to be considered ‘non-essential’ with the closure of national borders prompted me to reflect on what I had previously considered normality as a privileged migrant. I was deeply impressed by these individuals’ motivations to prioritize existential fulfilment and quality of life over career pursuits.

I analysed the interviews through thematic coding, narrative and interpretive analysis. I focused on the multifaceted experiences and existential implications inherent in the phenomenon of return and remote work, and related paced mobilities. I examine the importance of various mobilities, extending beyond return, and how some of these were deemed more essential than others at the personal level. I explore how mobility choices shape both personal and professional spheres. In the next section, I explore how the pandemic worked as a catalyst to switch from essential to existential mobilities: I delve into the meanings of the physical and existential move to a ‘better place’, and look at the spatial and temporal dimensions of the interplay between return mobilities and remote work.

## When life moves on to (or stays in) ‘a better place’

One of the prominent themes that emerged during my fieldwork centred on the newfound potential for individuals to work from any location, a trend that gained momentum in the wake of the pandemic. This potential held exceptional importance during the lockdown, when physical separations from familiar places, loved ones, and friends were amplified. Mobility regimes for those ‘normally’ considered as ‘privileged’ mobile people, who can supposedly move when and how they wish, were shaken. Some migrants faced the inability to travel to visit distant families, both for receiving or giving support in case of need. Even migrants experiencing no financial strain expressed concerns about childcare during the lockdown, or about attending the funerals of elderly parents if they died: Covid-19 served as a catalyst for return decisions that were already being considered before the pandemic (Mencutek, 2022 ). Returning to Italy, to one’s birth-town or home region, in association with the proliferation of online working during the pandemic, represents a particular way of taking advantage of the flexibility of remote working and overcoming this sense of insecurity. Unlike the conventional image of a digital nomadic lifestyle characterized by constant travel to different destinations, these returns are rooted in the desire to establish a stable and secure anchor in one’s place of origin. Respondents emphasized the desire to be in the ‘best place’ or to seek a sense of security during the pandemic. They expressed diverse motivations for return that included a desire to reconnect with family, particularly parents. Overcoming feelings of solitude, fulfilling aspirations, such as building a family or a home, were also pivotal factors. Sometimes, the idea of returning emerged during the solitude imposed by the lockdown itself: some faced uncertainty and sought comfort in the proximity of their loved ones, while others chose to leave behind the isolation of remote work and returned to share the lockdown period with their families.

For some, the thought of returning had crossed their minds previously, but it was the Covid-19 pandemic that convinced them in the end (Mencutek, 2022). Maria’s journey illustrates this. After more than three years of living abroad, she returned to Italy. The pandemic acted as a catalyst for Maria^5^, prompting her to make a decision that she had considered earlier but had not acted on. Reflecting on this, she says:

The pandemic influenced my choice of returning to Italy, both because of a sense of security, that feeling of being in your country, and because I started wondering ‘What am I missing by being away?’ I realized that I was a self-exiled because I was angry with my country. Then I started wondering: this anger that made me leave, in the end who gains and who loses [by living far away]? And maybe these questions without the pandemic … maybe I would have asked them myself anyway but maybe later. It is like the pandemic accelerated a lot of things.

Maria’s words indicate a profound sense of self-imposed isolation – a form of ‘exile’ and physical detachment she experienced when leaving Italy. Return is not always easy. Certain respondents acknowledge experiencing a sense of remoteness and unfamiliarity with specific local cultural norms at the place they return to. Maria reports a sense of disappointment regarding Italy, for its limited and precarious professional opportunities, a feeling and a socio-economic condition that made moving to another country for her career *essential* in the first place. Some other respondents admit that being single at a certain age and prioritizing career over family may not align with the social expectations imposed on women in certain southern villages. They may feel alien in relation to some of these local values, but respondents often emphasize and reflect on the transformation they underwent through migration and return, like Maria. She talked about how, during these years, she has realized that her geographical distance and the seemingly *essential* career mobility of the past had, in many ways, estranged her from her roots. She reconsidered the balance between work and life:

I no longer want to choose where to live based on my job. […] I have always been a very diligent person when it comes to work […] during the pandemic, I felt a sort of rejection towards work, as in work is work, and my life is my life. It is as if I saw a difference, a clear separation between what I do and who I am.

This change was particularly striking given her previous dedication to her career. Yet, during the pandemic, a new perspective took shape – a feeling of detachment from work as an all-consuming force, and a growing recognition that her life transcends her career. The pandemic acted as a catalyst, leading her to perceive work as a component of her life rather than its defining feature – a shift that prompted a deeper evaluation of her priorities and of which mobilities should be associated with them.

This shift in her thinking and feeling, which was also echoed by others, became particularly pronounced during the lockdown. It was especially poignant for those with family residing abroad, unable to travel or receive visits due to Italy’s early exposure to the pandemic. Interestingly, this change overshadowed concerns about precarity and job insecurity. In a way, the uncertainty surrounding personal and family well-being during the pandemic appeared to outweigh concerns related to work, or to lead to a need to negotiate with professional uncertainty (Mancinelli and Salazar, 2023). Some were even willing to take the risk of potentially losing their jobs so as to continue working remotely and stay in Italy, emphasizing the vital importance of physically being in a place that offered a sense of security during such challenging times.

Gaia is a 35-year-old woman with children. To facilitate her move back from abroad, Gaia had to undergo a significant transformation in her work. Prior to her return, she had already transitioned to a more desk-based job, but she recognized the need to make additional adjustments to ensure it could be performed remotely. Gaia talked about how she combines her work with *essential* occasional trips to the workplace for meetings, reshaping her work arrangements to accommodate life and vice versa, and balancing the requirement to be physically present at work with more personal obligations: online and corporeal mobility here coexist, with the former complementing rather than substituting for the latter (Storme et al., 2017). For Gaia and other women like her, with family and children, the impact of the pandemic was important, especially in view of childcare arrangements and the distance from the extended family:

with the children, we no longer had grandparents who could come here […] whereas before I was used to having the grandparents more or less every two months […] it started a bit this sense of lack, of family, of help, we rethought a bit of everything … the pandemic gave us some food for thought.

Workers like Gaia were allowed to work from home during the lockdown, a form of privileged immobility and a form of capital in comparison to others (Salazar, 2021b). Home-working is connected with forms of immobility and mobility other than physical ones, such as professional and mental mobilities^3^ (Daniel et al., 2018). Home, while potentially creating a sense of isolation for some, may also represent a comfortable space for reflection and creativity, and for focusing on work as well as for reaching more people online, for example. Working remotely also allows people to spend time with family, focusing on hobbies (e.g. cooking, running, walking) and personal life, so as to have a sense that life is moving somewhere better (Cangià et al., 2023). However, certain mobilities (family visits, for example) were also banned as a result of the closure of national borders: they became non-essential from a governmental point of view, while acquiring a major importance for the respondents. The distance from extended family and related immobility represent the criteria through which respondents remade their sense of the course of their life as an existential movement to something ‘better’ (Hage, 2005), to a ‘good life’, and to have ‘control’ over it (Salazar, 2021a).

Paola (36 years old) recently returned to her hometown in Sicily after spending more than four years in different places around the world. Her journey back to her past was prompted by a deep introspection that took root during the lockdown period, with the various restrictions in the ‘normal’ daily life:

with the pandemic you have found yourself, good or bad … when the whole entertainment system collapses and it is you with your choices … you don’t have vacation … you must ask yourself what it is that you really want.… You start seeing the importance of the quality of life, of mental health, of human relationships, let’s say these were all things on which I have reflected when I decided to return the first time.

Paola’s experience, like that of others, reflects a renewed proximity to an envisioned future – a life that aligns with her aspirations. This doesn’t imply a disregard for her career, but rather a re-evaluation of the types of mobility she engages in to balance work and life. The opportunities offered by remote work take on a special significance here. It allows her to pursue professional endeavours while relocating to places that resonate with everyday rhythms. This shift offers time that can be dedicated to personal passions and embraced as an opportunity for slower modes of mobility. Paola continues:

Now [that I live in Sicily] the quality of life is unbeatable because … if I want to be alone, I’m alone, otherwise, I can go out and I have my network of people to see. I have my parents nearby, the sea.… For me these things matter.… I take a lot of care of myself now, much more than I could do in a big city where there is commuting and a much more hectic life.

I now look more deeply into this temporal dimension, with the exploration of variously paced im/mobilities.

## ‘Smart’ working and ‘quiet’ movement: pacing im/mobilities during the pandemic

Other forms of immobility emerged through the combination of people returning and the opportunities to work remotely. Some respondents moved from cities to more rural environments, that is, to remote places, a movement that helps people find a new sense of time and a new pace or rhythm of life – to walk, to take breaks from work, and to go out and enjoy the environment (Cook, 2020). Ornella, a woman in her forties who recently moved back from North Italy to her hometown in the South, vividly captures this transformation. During the lockdown, a period that wiped out many taken-for-granted routines, Ornella found herself gravitating towards a more contemplative approach to life, appreciating the beauty of touristic spots and her hometown. She says:

I took advantage of this time to also stay a bit isolated, to walk, to rediscover places, neighbourhoods. I mean, everywhere you turn oozes history, things to see, things to discover.… Here I feel extremely stimulated … maybe one might think that a big city has more opportunities. Yes, perhaps professionally. However, from a life and sociocultural perspective, I feel much more fulfilled here.… the fact that I can work from here remotely, it is even better, because truly remote working gives you a time management that you wouldn’t normally have if you commute to work.

These words reflect Ornella’s preference for a slower, more culturally enriching lifestyle in a smaller town with historical significance. Despite the professional opportunities that a big city may offer, she feels more fulfilled now she says. The flexibility of remote work in remote places enhances this work–life balance, highlighting the importance of well-being and cultural experiences in achieving personal satisfaction. Like Ornella, most respondents reported a renewed sense of joy in daily activities that they stopped experiencing while living elsewhere. Walks by the sea, visits to their town’s historic and touristic places, or the simple pleasures of cafes, restaurants, and conversations in their native language were rediscovered. This sense of intimacy with the cultural fabric of the ‘South’ awakened a deeper connection with the place of return.

Iacopo, in his forties, made the journey back to the South from the city of Rome, where his company has its headquarters, during the latter part of the lockdown, and went on to stay there for a year. For him, being close to the beach and living in a small village amplifies the positive aspects of living in a less populated area. The rhythms of life shift dramatically, allowing for moments of relaxation, time to swim in the sea, as opposed to the daily commuting between home and work in a big city. Iacopo describes this shift:

That changed my way of life, because working from home at six o’clock you close your computer, see your friends, go for a walk or in winter you go for a beer, in summer you go to the beach … you do everything more calmly. In Rome, instead, you must organize yourself simply to go to the supermarket, you cannot just say ‘I’ll go down and get bread’ because if you go to the supermarket, you lose time. Here grocery shopping takes five minutes instead … [Within] Five hundred metres I could get everywhere.

Iacopo made a conscious choice to return to his small coastal hometown, seeking a slower pace of life. His decision to work remotely and live in a less populated area is a response to the ‘overdrive’ pace of urban life (Amit and Salazar, 2020). His move allows him to break away from the excessive activity and speed associated with living in a big city. Some individuals may yearn for a slower pace, seeking a deeper connection with their environment, community, and personal well-being. Iacopo’s lifestyle shift aligns with the ethos of ‘slow movements’ (Amit and Salazar, 2020), as he seeks to evoke ideas about the proper pace of a ‘good life’ that is more sustainable and fulfilling. Respondents like Iacopo indeed report a sense of taking back control of their life, something that had gone missing with previous migration to destinations for work.

This ‘quiet movement’, as Tommaso defined it, represents one of the main aspects defining the transformations occurred in these people’s lives after the pandemic. The experience of Tommaso, who also relocated from Rome to the South, well illustrates how to regain time by moving to a small town. This slow pace, set against the backdrop of work and the rapid pace of city life, defines Tommaso’s feeling of being at home:

I got used to this quiet movement, I mean here we move in ten minutes. One thing about Rome that I hate instead is moving around in the city.… Maybe there is a certain routine life here that might seem boring, anyway if you feel fine where you are and where you live according to your own rhythm, for me that is home.… Here the hands of the clock run slower.… I can do so many things. Times are short, distances are very short.

For some, the time previously consumed by commuting or business travel is now viewed as ‘time gained’, facilitating alternative and more important forms of mobility, such as sports, family visits, or tourism (Cook, 2020). With Covid-19, new norms and expectations surrounding remote work became battlegrounds for negotiation between employees and employers (Selmer et al., 2021). Return often entails discussions with employers about the feasibility of remote work, reshaping mobility patterns and fostering a sense of connection to specific places – an essential strategy to delineate the boundaries between work and personal life (Cangià, 2023b).

These people’s focus on reclaiming time, embracing slower movements, and staying close to meaningful places reflects changes in contemporary work patterns: the pandemic prompted a need to re-evaluate work–life boundaries (Cook, 2020). As individuals like Iacopo and Tommaso return to their old hometowns, they simultaneously renegotiate the relationship between work and personal life. Return is thus a form of existential mobility, ‘essential’ for the cultivation of a better life in a familiar environment, where personal history and future aspirations may converge in the present. Movement isn’t just physical but a deep transformation of one’s connection to self, community and place.

Return, here, is not merely about the freedom to travel to various locales at will; instead it represents an existential shift, and is driven by a different sentiment than that usually associated with ‘digital nomadism’. Beyond mere movement, return signifies a commitment to creating a more fulfilling life through alternative mobilities in an environment where the past, present and future seamlessly intertwine. Combined with remote work, return also functions as a strategy to navigate the growing economic instability in Italy, and improve the conditions in the southern part of the country. It may offer a chance to counterbalance these challenges by tapping into better economic chances in northern regions or countries, where work is located – all this is made possible through the flexibility and accessibility of remote work (Mancinelli and Salazar, 2023). In this sense, ‘we are always South to some other place’, as one of the founders of the association South-Working claimed. Some respondents told how they organized community hubs (*presidi di comunità*), aiming to attract tourists to explore Sicily while appreciating a remote-working vacation in these locations and enjoying slower mobilities. Tommaso tells me about his project:

Through our network and established channels over time, we’ve invited people [to come and work from Sicily] […]the majority came between May and June, and we provided co-working spaces for work, accommodations, and … organized activities where they could enjoy the local area, essentially offering them a true vacation experience.

In the spirit of the idea of *workation*,^4^ a concept already familiar to many digital nomads, which involves planning a trip to a tourist destination while working, here the boundaries between the experiences of these people’s return, digital nomadism and tourism mobilities, between ‘host’ and ‘guest’ as well as between different mobility temporalities, become blurred (Bloch, 2020; Cook, 2020).

## Remote work and return in pandemic times: overcoming categories and regimes of mobilities

The pandemic has brought about a potential long-term shift at the organizational level, paving the way for the reconfiguration of work structures and locations within various professional domains (Liebowitz, 2020). The once niche phenomenon of remote work, previously associated with digital nomadism, has expanded to include a broader spectrum of people (Cook, 2020). Global mobility and digitalization have enabled individuals to make choices about their travel, living arrangements, and work locations and rhythms. Conversely, remote work has also evolved into a more lasting arrangement for certain people. This newfound flexibility in work arrangements has made return more feasible for participants in this study, contributing to a multifaceted transformation in the way they work, live and move.

Professional mobility has long been characterized by individuals relocating for career (Montulet and Mincke, 2019). However, the pandemic introduced new dynamics. It has acted as a catalyst, accelerating the shift towards work increasingly coming to people, as opposed to the traditional approach of relocating people to their workplaces. The global trend of work migrating to individuals, coupled with the flexibility of digital mobility, has reshaped the choices some people are able to make about where they work and reside (Hermann and Paris, 2020). This has led to a significant reconfiguration of the boundaries between work and life. Moving work to people via the digitalization can enable individuals to decide how much they want to travel, and to choose their preferred locations for living and working (Selmer et al., 2021): ‘I no longer want to choose where I live based on my job,’ says Maria.

People who were accustomed to relocating for their career started to deal with challenges such as their work–life balance and worries about the well-being of distant family members during the crisis. Elena, who moved back to Italy from a foreign country, vividly recalls the fear she felt seeing her country and family hit by the pandemic:

[If] all these things had happened with me from a distance it would have been worse, I would have had, let’s say, a sense of loss of control, which made it a bit intolerable for me to be away.… That is this perception of remoteness, that you could not physically go to the hospital to see anyone.

This transformation highlights the influence of unprecedented events like a pandemic on the evolution of work dynamics and mobility patterns. Workers’ return – to their country or hometown –and family visits represent some of these mobility patterns: the event of the pandemic shifted the understanding of what was considered as essential mobility to a more existential feature of movement, and represented a time when life-related mobilities became in fact more ‘essential’ for people than work-related mobilities. Personal mobilities to visit family were exactly the forms of mobility considered non-essential by the governments during the crisis. On a more personal level, respondents seem to shift priorities from essential mobilities related to their career (moving to another place where job opportunities seem better and greater in number), to an essential immobility related to family and personal life. Other forms of mobility, such as intermittent commutes, remote work and mental mobility become essential: each respondent has crafted their unique strategy to blend various mobilities, such as short-term travel and intermittent commuting, tourism or other forms of urban mobilities, online and corporeal mobility to the workplace, in support of their choice to return. In this sense, the pandemic has not only accelerated the adoption of remote work but also expanded the concept beyond the traditional association with digital nomadism towards forms of ‘rooted digitalism’, where the sense of home and place-making become prominent (Cangià et al., 2023). It came to encompass a broader range of im/mobile people, challenging the conventional understanding of who can engage in remote work – and when.

## Conclusions

This study has practical implications for conducting fieldwork among remote workers, showing that researchers need to account for the diverse forms of mobility that have emerged in the post-pandemic world. Theoretically, it challenges the traditional concepts of ‘essential’ and other more ‘existential’ mobilities such as digital nomadism, suggesting that returnees and remote workers occupy a new space where mobility is flexible, shifting, and intertwined with both personal and professional life trajectories. Respondents emphasized the importance of shifting the popular image of return, and stressed that return should not be viewed as a move back to the past, but rather as a fresh opportunity for a transformative future. They suggested we should move away from the dichotomy between return as a failure and return as a success (de Haas et al., 2015). I aimed to do justice to their perspectives by portraying their return experiences in relation to the re-making of life trajectories and the practice of mobilities in the aftermath of the pandemic. The practice and imaginings of mobility are inextricably intertwined with the making and imperatives of career (Cangià, 2021; Le Feuvre et al., 2022) and with the experience of time (Amit and Salazar, 2020). Return intertwined with remote work is more than a return to the past ‘home’ but becomes a creative future-oriented project (Markowitz and Stefansson, 2004), a return to various places, ‘real, virtual, imagined, desired, forced or denied’ (King and Christou, 2011: 453).

Mobility includes various forms of movement associated with return, challenging the neat separation between ‘essential’ and ‘non-essential’ mobilities, work and other lifestyle mobilities, between ‘returnees’ and ‘migrants’, immobile ‘host’ and mobile ‘guest’ (Bloch, 2020). Respondents indeed engage in return. However, they also continue working at a distance, participating in various forms of mobilities such as commuting, work travels, visiting family and friends, engaging in tourism or in other urban mobilities, while conducting work through online digital platforms, transporting themselves mentally to the workplace or connecting with colleagues. Mobilities can be essential in one aspect of life but less so in others, and their relative importance may shift, becoming essential at some points in life and then non-essential at others. Despite being returnees in one sense, respondents remain on the move. They choose to prioritize life over work, yet they remain attentive to their career, finding opportunities for continued growth through remote work and intermittent travels. They are not merely ‘guests’ in their homeplace, but do not entirely transform into ‘hosts’ upon their return, preserving a complex connection with the sense of coming ‘home’ as they remember it.

These experiences of return help us reflect on common understandings of essential movement. The pandemic has blurred the lines between conventional mobility categories and regimes, disrupting the distinction between ‘essential’ and ‘non-essential’ mobility, as well as challenging the boundaries separating returnees, migrants, digital nomads, tourists, host communities, and other im/mobile individuals. It not only accelerated the acceptance of remote work but also broadened its scope beyond the confines of traditional digital nomadism, embracing a wider array of mobile individuals and challenging conventional notions of who can work remotely, under what circumstances, and with what implications. Further studies should assess the impact of these transformations beyond the pandemic, to understand enduring changes in mobility patterns.

It is worth noting that individuals with families are less common in this phenomenon. In respondents’ narrations, however, gender dynamics are not explicitly discussed but still latent. Exploring how women, particularly those with family, navigate the intersection of remote work, return and caregiving duties could add depth to the analysis. It could also indicate major inequalities in terms of childcare infrastructure between ‘North’ and ‘South’. Further studies should incorporate a gender perspective and provide a nuanced understanding of how returnees’ experiences, motivations, and negotiations with remote work might differ based on gender roles and family arrangements, as well as on the phase of one’s life-course (Le Feuvre et al., 2022).

The capacity to revise values and life priorities amidst job uncertainty during the pandemic reveals also how immobility – defined as the capacity to stay and not move – represents a form of capital and a privilege for some workers (Adey et al., 2021; Salazar, 2021a). It is crucial to acknowledge that not everyone had the opportunity to return after the pandemic, and to assess the extent to which remote work represents a privilege in this context (Mancinelli, 2020). The conditions under which people could return vary significantly, as the ‘South’ in South-Working can partly suggest. These variations highlight the increasing inequalities in mobility and work experiences evident during and after Covid-19, and evidence the need for a more nuanced understanding of the complexities surrounding mobilities and digitalism in the post-pandemic era.

## Notes

1 ‘Digitalism’ refers here to the digitalization of work, the increasing practice of digital work enabled by technological tools and digital devices. The term does not simply refer to the fact that people now use technologies more than before, but also to the routines and condition of living with the digital in different spheres of life (Bowen and Giannini, 2014; Cangià et al., 2023).

2 Including migration, return, tourism, digital nomadism, lifestyle, professional, and existential mobilities.

3 Mental mobility refers to a cognitive flexibility in navigating and interpreting information and ideas (Daniel et al., 2018).

4 The topic of *workation* will not be further examined here. However, it represents another aspect of remote work and its intersection with return mobilities.

5 All names are pseudonyms.
